# Increased testicular insulin-like growth factor 1 is associated with gonadal activation by recombinant growth hormone in immature rats

**DOI:** 10.1186/s12958-022-00944-z

**Published:** 2022-04-23

**Authors:** Yang Xu, Chang Yong Han, Mi Jung Park, Myung Chan Gye

**Affiliations:** 1 Department of Life Science and Institute for Natural Sciences, Hanyang University, Seoul, 04760 Korea; 2 Department of Pediatrics, Sanggye Paik Hospital, Inje University College of Medicine, Seoul, 01757 Korea

**Keywords:** Recombinant human growth hormone, Spermatogenesis, Steroidogenesis, Insulin-like growth factor-1, Kisspeptin, Gonadotropin-releasing hormone, Rats

## Abstract

**Background:**

In children, recombinant human growth hormone (rhGH) therapy for treatment of short stature has raised concerns of the early onset of puberty. Puberty is initiated by the activation of the hypothalamus-pituitary-gonad axis. Insulin-like growth factor-1 (IGF1) has been known to mediate physiologic effects of GH. To understand the mechanism of precocious sexual maturation following prepubertal GH therapy, the effects of rhGH on the hypothalamus-pituitary-gonad axis were examined in the immature male rats.

**Methods:**

Immature male rats were given by daily injection of rhGH (1 or 2 IU/kg) from postnatal day (PND) 21 to PND 23 or 30. The effects of rhGH on kisspeptin-GnRH-LH system in the hypothalamus-pituitary axis, systemic and testicular IGF1, spermatogenesis, steroidogenesis, and circulating testosterone levels were examined. The effects of rhGH on the IGF1 expression and steroidogenesis were examined in progenitor LCs *in vitro*.

**Results:**

Testicular steroidogenic pathway and spermatogenesis marker mRNA levels, number and size of 17β-hydroxysteroid dehydrogenase (+) LCs, and blood testosterone levels of rhGH rats were significantly higher than those of controls on PNDs 24 and 31. Hypothalamic *Kiss1* and *Gnrh1* mRNA of rhGH rats were significantly higher than those of controls on PND 24, indicating early activation of hypothalamic kisspeptin-GnRH neurons by rhGH. Hypothalamic *Igf1* mRNA levels of rhGH rats were significantly higher than those of controls on PND 24 but significantly lower than those of controls on PND 31. Testicular *Igf1* mRNA levels were significantly higher in rhGH rats than in the controls on PNDs 24 and 31 whereas circulating IGF1 levels were not. In progenitor LCs, rhGH significantly increased *Igf1* and steroidogenic pathway mRNA levels and testosterone production.

**Conclusions:**

Local increases in testicular IGF1 might be an important mediator of gonadal maturation via activation of LCs steroidogenesis in immature rats given rhGH.

**Supplementary Information:**

The online version contains supplementary material available at 10.1186/s12958-022-00944-z.

## Background

Puberty is initiated by the activation of the hypothalamus-pituitary-gonad axis (HPG axis). In the hypothalamus, kisspeptin activates gonadotropin-releasing hormone (GnRH) secretion, resulting in luteinizing hormone (LH) secretion in the pituitary gland, steroidogenesis and gametogenesis in the gonads, and the onset of puberty [1–3]. As a neuromodulator, kisspeptin not only conveys the modulatory actions of sex steroids to GnRH neurons but also directly stimulates LH secretion in the pituitary gland [4]. Growth hormone (GH) has been shown to play a role in the onset of puberty, and it regulates the expression of steroidogenic acute regulatory protein (STAR) and steroidogenesis in progenitor Leydig cells (PLCs) [5]. GH-receptor knockout mice and GH-deficient mice show delayed puberty [6, 7]. GH therapy normalizes the progression of puberty in GH-deficient mice [8, 9]. Most of the physiologic effects of GH on puberty have been explained by the actions of insulin-like growth factor-1 (IGF1) [10–12]. IGF1 is primarily produced in the liver, but it is also synthesized by almost all tissues, including the testes [13, 14]. In mammals, including humans, circulating IGF1 levels increase at the onset of puberty, and the administration of IGF1 advances the process of puberty [15, 16]. In Laron syndrome patients, who have congenital IGF1 deficiency, the administration of IGF1 restores sexual maturation [17]. In immature animals, IGF1 can activate the brain-gonadal axis and contributes to the timing of puberty in males and females by affecting GnRH neurons and possibly kisspeptin neurons in the hypothalamus [18]. Recombinant human growth hormone (rhGH) therapy has been introduced as a treatment for a variety of conditions associated with short stature, such as GH deficiency [19], Turner syndrome [20], chronic renal failure [21], short stature homeobox gene [22, 23], and Noonan syndrome [24]. However, there are concerns that it can induce skeletal maturation and the early onset of puberty, though some studies have reported that rhGH therapy did not correlate with early puberty onset [3, 25, 26]. To understand the mechanism by which rhGH could stimulate the early onset of puberty in males, changes in hypothalamic kisspeptin, GnRH and IGF1 levels, pituitary and circulating LH, spermatogenesis, testicular steroidogenesis, and IGF1 in the circulation, liver, and testes were examined in immature rats following prepubertal rhGH treatment.

## Materials and methods

### Animals and tissue sampling

Immature male Sprague Dawley (SD) rats on postnatal day (PND) 14 were purchased from Daehan Biolink (Deajeon, Korea). The animals were randomized into three groups and subcutaneously injected with rhGH (1 or 2 IU/kg) or 0.1 mL of saline every morning at 11 a.m. from PND 21 to PND 23 or 30. After rhGH treatment, animals were sacrificed by CO_2_ asphyxiation on PND 24 or 31. Blood was collected by cardiac puncture and prepared for serum. The testes, epididymis, prostate, preputial glands, seminal vesicle, and brain were dissected and weighed. The hypothalamus and pituitary gland were further dissected from the brain. Organs were subjected to histological and gene-expression analyses.

### Real-time reverse transcription-polymerase chain reaction (RT-qPCR)

Dissected tissues were subjected to total RNA extraction using TRI reagent (Molecular Research Center, Cincinnati, OH, USA) according to the manufacturer’s instructions. Testicular steroidogenic and spermatogenic markers, and *Igf1* mRNA levels were analyzed by RT-qPCR. In the hypothalamus and pituitary gland, *Igf1*, *KiSS-1 metastasis suppressor* (*Kiss1*), *gonadotropin Releasing hormone 1* (*Gnrh1*), and *luteinizing hormone beta polypeptide* (*Lhb*) mRNA levels were analyzed. *Ribosomal protein L7* (*Rpl7*) was used as an endogenous control. The primer sequences and PCR conditions are summarized in Table 1. RT-qPCR was performed using an SYBR Premix EX Taq kit according to the manufacturer’s instructions (Takara, Shiga, Japan). Relative mRNA levels were calculated using the comparative 2^−ΔΔCt^ method [27].

**Table 1:** Primer sequences used in RT-qPCR analyses

Genes	Primer sequences (5′–3′)		Size	Ann. temp.	GenBank ID
*Igf1*	F	tacctggcactctgcttgct	194	64	NM_001082477
	R	cggaagcaacactcatccac			
*Lhcgr*	F	ctcacctatctccctgtcaa	365	64	NM_012978
	R	acagactcgttattcatccc			
*Sf1*	F	cgccaggagtttgtctgtct	185	67	NM_001191099
	R	acctccaccaggcacaatag			
*Star*	F	aaccaggaaggctggaagaa	123	64	NM_031558.3
	R	tctgtccatgggctggtcta			
*Cyp11a1*	F	ttgcctttgagtccatcacc	187	64	NM_017286.2
	R	gcatggtccttccaggtctt			
*Cyp17a1*	F	aacgttgactccagcattgg	163	60	NM_012753.2
	R	gcgtgggtgtaatgagatgg			
*Hsd3b1*	F	gcattaaccccactcccact	146	60	NM_001007719.3
	R	ggaccctgacctccttcaga			
*Hsd17b3*	F	gtccctggcctctttacagc	191	64	NM_008291
	R	tttaacaaactcatcggcgg			
*Cyp19a1*	F	tatccggaggtggaaacagc	181	60	NM_017085
	R	cgtcaatcacgtcatcctcc			
*Tnp1*	F	gatgcaagtcgcaattaccg	184	67	NM_017056.2
	R	ccgaatttcgtcacaactgg			
*Tnp2*	F	gaagaccttggaagggaaagtg	150	64	NM_017057.2
	R	tggctatctccttttgggat			
*Prm2*	F	tatgggaggacagaaagggg	157	58	NM_012873
	R	tcctccttcgggatcttctg			
*Kiss1*	F	agctgctgcttctcctctgt	152	64	NM_181692
	R	aggcttgctctctgcatacc			
*Gnrh1*	F	ccgctgttgttctgttgact	150	64	NM_012767
	R	ggggttctgccatttgatcc			
*Lhb*	F	tcccaggactcaaccaatga	112	60	NM_001082477
	R	tggttagaacacctgctggc			
*Rpl7*	F	tcaatggagtaagcccaaag	246	60	NM_011291
	R	caagagaccgagcaatcaag			

### Histological and immunohistochemical analysis

Hematoxylin-Eosin staining, and image analyses of testes was carried out according to previous study [3]. At least eight testes at each rhGH dosage and more than 10 seminiferous tubules per testis were measured to determine the seminiferous tubule diameter and luminal area. For immunohistochemical labeling of 17β-hydroxysteroid dehydrogenase (HSD17B) Bouin’s solution-fixed testes section were deparaffinized, rehydrated, blocked by incubation with 1% rabbit serum in phosphate buffered saline (PBS) and incubated with rabbit anti-HSD17B antibody (sc-31,620, Santa Cruz Biotechnology, Dallas, TX) diluted to 4 μg/mL in 1% rabbit serum in PBS in a humidified chamber overnight at 4 °C. In the negative control, normal rabbit IgG (ab172730, Abcam, Cambridge, UK) diluted to 4 μg/mL in 1% rabbit serum in PBS was used instead of the primary antibodies. After washing in PBS, horseradish peroxidase-conjugated goat anti-rabbit IgG (ab175470, Abcam) diluted 1:200 in 1% rabbit serum in PBS was applied, and the slides were incubated for 30 min at room temperature. After being washed in PBS, the slides were stained with hematoxylin and eosin, mounted with Canada balsam (03984, Sigma-Aldrich), and observed with an epifluorescence microscope (IX71, Olympus, Tokyo, Japan) equipped with a digital imaging system (DP71, Olympus).

### Immunofluorescence labeling of HSD3B in rat testes and Leydig cells

Paraformaldehyde (PFA, 4%)-fixed testis sections or isolated Leydig cells (LCs) were subjected to immunofluorescence staining of 3β-hydroxysteroid dehydrogenase (HSD3B). Briefly, sections on poly-L-lysine coated slides were deparaffinized. Isolated LCs were fixed by 4% PFA and washed with PBS. Testis sections and LCs were blocked with 1.5% normal donkey serum in PBS for 30 min, and then incubated overnight at 4 °C with rabbit anti-HSD3B antibody (sc-30,820; Santa Cruz Biotechnology) at 1:100 in blocking solution. As a negative control, normal rabbit IgG (ab172730, Abcam) replaced the primary antibody. After washing in PBS, slides were incubated in a 1:200 dilution of donkey polyclonal antibody to rabbit IgG Alexa Fluor (ab175692, Abcam) in blocking solution for 30 min. After 4′,6-diamidino-2-phenylindole (DAPI; 32,670, Sigma-Aldrich) staining, observation and photography were conducted using an epifluorescence microscope system.

### *In vitro* rhGH treatment of rat LC primary culture

The decapsulated testes of PND 21 male SD rats were washed with PBS and incubated with collagenase (0.25 mg/mL)-RPMI medium (11,875,093, Gibco, Dublin, Ireland) for 20 min. The interstitial cells from the supernatant were washed 2 times with RPMI medium. To obtain purified LCs, 1 mL of the interstitial cells was loaded at the top of a 36–60% (v/v; 2 mL each) Percoll (P1644, Sigma-Aldrich) gradient. After centrifugation at 800 g for 25 min, four visible bands of testicular cells were obtained, with highly purified LCs found in the third band from the top, corresponding to a 38–52% (v/v) Percoll concentration. LCs were collected, washed twice with RPMI and resuspended in RPMI with 10% fetal bovine serum (10,270,106, Gibco) and 1 X Antibiotic-Antimycotic (15,240,062, Gibco). Purity of LCs was assessed by HSD3B immunofluorescence. For rhGH treatment, purified LCs were plated in 35-mm culture dishes at 10^5^ cells/mL and incubated at 37 °C in a humidified atmosphere of 5% CO_2_. After 24 h, the medium was replaced with RPMI with 0, 10, and 50 μg/L rhGH and 10 μg/L human chorionic gonadotropin (hCG). After 48 h treatment, the spent media were collected and subjected to enzyme-linked immunosorbent assay (ELISA) to determine testosterone concentration. The mRNA expression of the rhGH-treated LCs was analyzed by RT-qPCR.

### ELISA for circulating testosterone, LH, and IGF1 levels

Total serum testosterone (T), IGF1, and LH concentrations of rats were measured on PNDs 24 and 31 by ELISA using testosterone and IGF1 ELISA kits (Demeditec Diagnostics, Kiel-Wellsee, Germany) and LH kit (Shibayagi, Shibukawa, Japan) according to the manufacturers’ instructions.

### Statistical analysis

Data were presented as box plots. Statistical calculations were performed using Mann-Whitney U test (IBLM SPSS Statistics 21.0, IBM Corporation, Armonk, NY). A *p*-value of less than 0.05 was considered statistically significant.

## Results

### Changes in testis weight, seminiferous tubule histology, and mRNA levels of spermatogenic marker genes

**Fig. 1:**
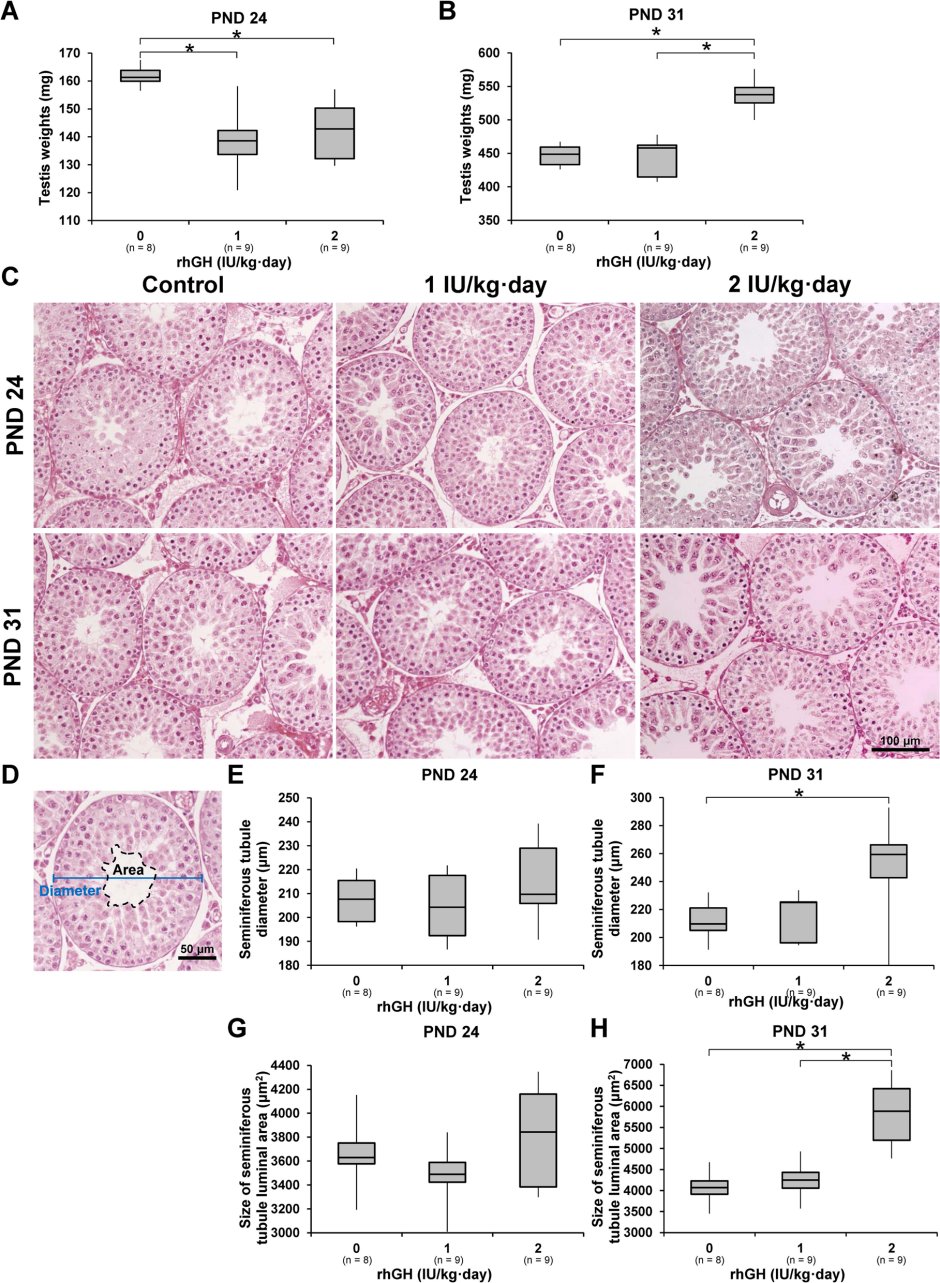
The effects of rhGH on testis weight, histology, seminiferous tubule diameter, and size of the luminal area in immature rats. **A**, **B** The testis weights in immature rats given rhGH. **C** Hematoxylin and eosin staining of testes from immature rats. **D** Image analysis of seminiferous tubule diameter (blue line) and luminal area (dotted line). **E**, **F** Diameter of seminiferous tubules from immature rats. **G**, **H** Size of the luminal area of seminiferous tubules from immature rats. *, significantly different from control rats by Mann-Whitney U test at *p* < 0.05

**Fig. 2:**
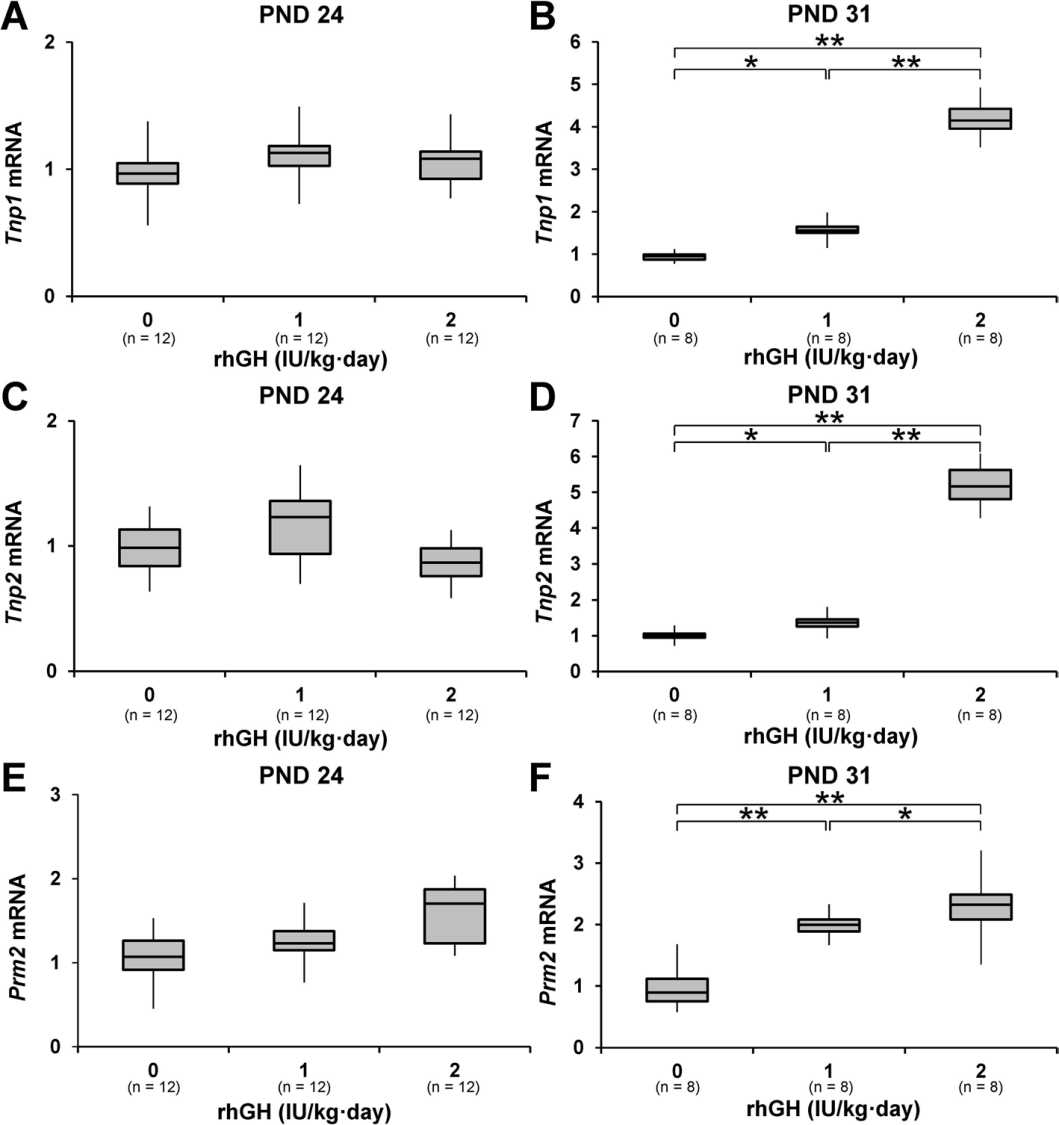
The effect of rhGH on spermatogenic marker mRNA in immature rats. Testicular *Tnp1* (**A**, **B**), *Tnp2* (**C**, **D**), and *Prm2* (**E**, **F**) mRNA levels were examined using RT-qPCR. * and **, significantly different from control rats by Mann-Whitney U test at *p* < 0.05 and 0.01, respectively

On PND 24, following three daily administrations of rhGH, the testis weights of the 1 and 2 IU/kg rhGH rats were significantly lower than those of the control rats. However, on PND 31, following 10 days administrations of rhGH, the testis weights of the 2 IU/kg rhGH rats were significantly higher than those of the control and 1 IU/kg rhGH rats. In the testis histology examination, the seminiferous tubule diameter was significantly increased in the 2 IU/kg rhGH rats on PND 31 but not in the 1 IU/kg rhGH rats. The seminiferous tubule luminal areas of 2 IU/kg rhGH rats on PND 31 were significantly increased compared to those of controls and at 1 IU/kg rhGH rats. No significant difference in seminiferous tubule diameter or luminal area was observed between the control and 1 or 2 IU/kg rhGH rats on PND 24 (Fig. 1; Supplementary Fig. 1). The testicular *Transition protein 1* (*Tnp1*), *Transition protein 2* (*Tnp2*), and *Protamine 2* (*Prm2*) mRNA levels of 1 and 2 IU/kg rhGH rats were significantly higher than those of the controls on PND 31 but not on PND 24. The *Tnp1*, *Tnp2*, and *Prm2* mRNA levels of 2 IU/kg rhGH rats were significantly higher than 1 IU/kg rhGH rats on PND 31 (Fig. 2).

### Changes in the number and size of LCs, testicular steroidogenic pathway gene mRNA levels, and blood testosterone levels

**Fig. 3:**
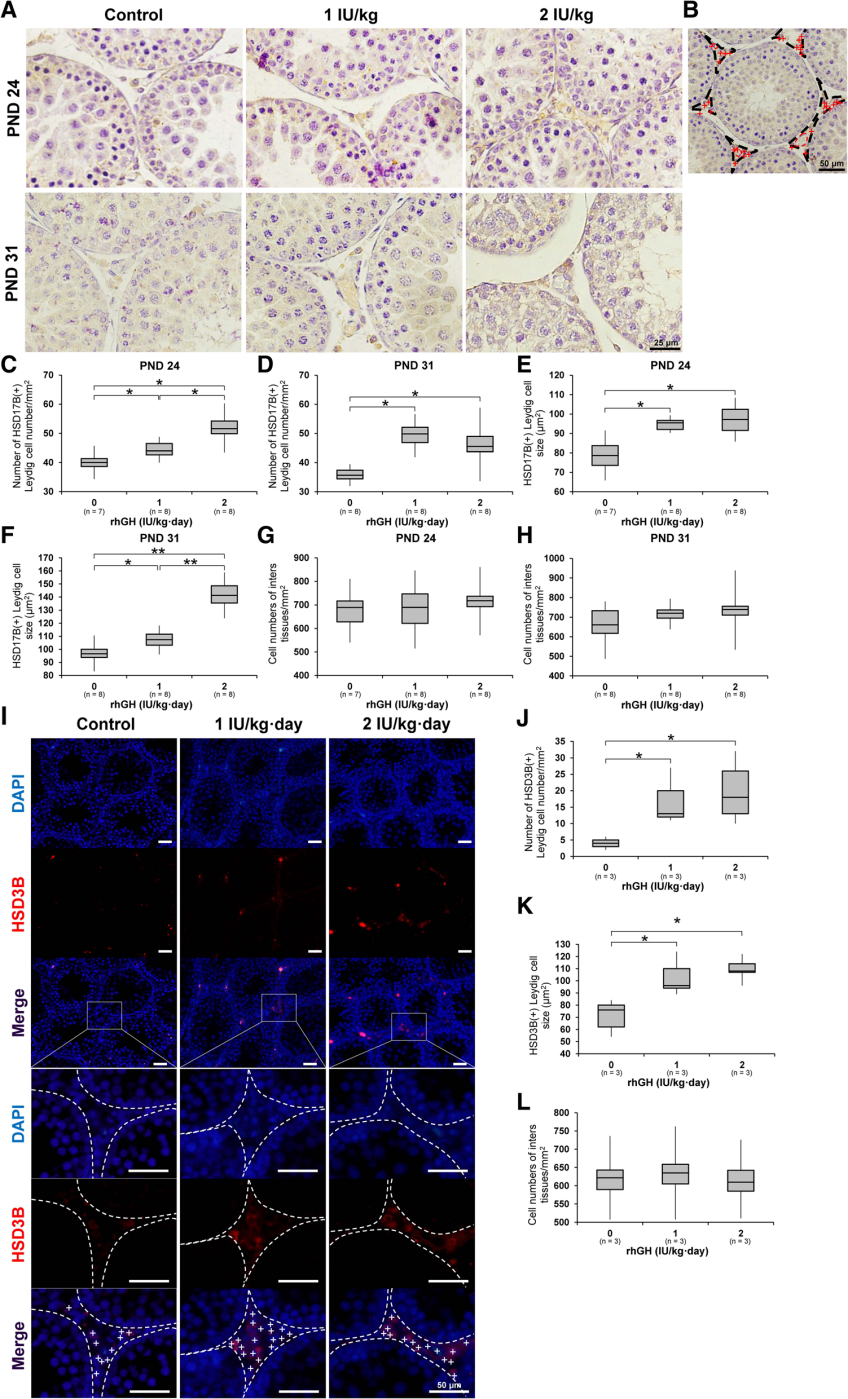
The effects of rhGH on HSD3B(+) and HSD17B(+) LCs in immature rat testes. **A** Immunohistochemistry of HSD17B in immature rat testes on PNDs 24 and 31. **B** Image analysis of HSD17B(+) LCs. Red cross, nuclei of LCs; black dotted line, boundary of HSD17B(+) LCs; red dotted line, boundary of blood vessels. **C**, **D** Number of HSD17B(+) LCs in each unit area (1 mm^2^) of the testes. **E**, **F** Mean size of HSD17B(+) LCs. **G**, **H** Total number of HSD17B(+) interstitial cells in the testes of immature rats on PNDs 24 and 31. **I** Immunofluorescence of HSD3B in immature rat testes on PND 24. White cross, nuclei of LCs; white dotted line, boundary of HSD3B(+) LCs; white box, area shown in the high magnification photograph. **J** Number of HSD3B(+) LCs in each unit area (1 mm^2^) of the testes. **K** Mean size of HSD3B(+) LCs. **L** Total number of HSD3B(+) interstitial cells in the testes of immature rats on PND 24. * and **, significantly different from control rats by Mann-Whitney U test at *p* < 0.05 and 0.01, respectively

**Fig. 4:**
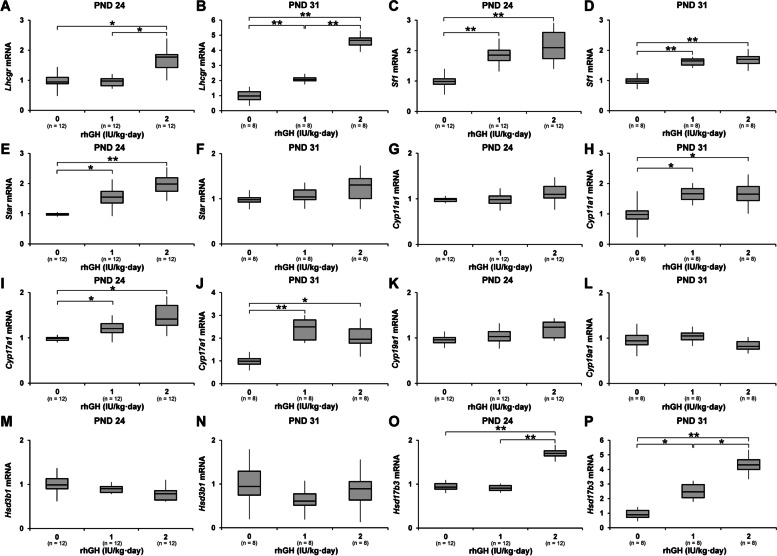
Effects of rhGH on the expression of steroidogenic pathway gene mRNA in immature male rats. RT-qPCR analysis of *Lhcgr* (**A**, **B**), *Sf1* (**C**, **D**), *Star* (**E**, **F**), *Cyp11a1* (**G**, **H**), *Cyp17a1* (**I**, **J**), *Cyp19a1* (**K**, **L**), *Hsd3b1* (**M**, **N**), and *Hsd17b3* (**O**, **P**) mRNA levels in the testes on PNDs 24 and 31. * and **, significantly different from control rats by Mann-Whitney U test at *p* < 0.05 and 0.01, respectively

**Fig. 5:**
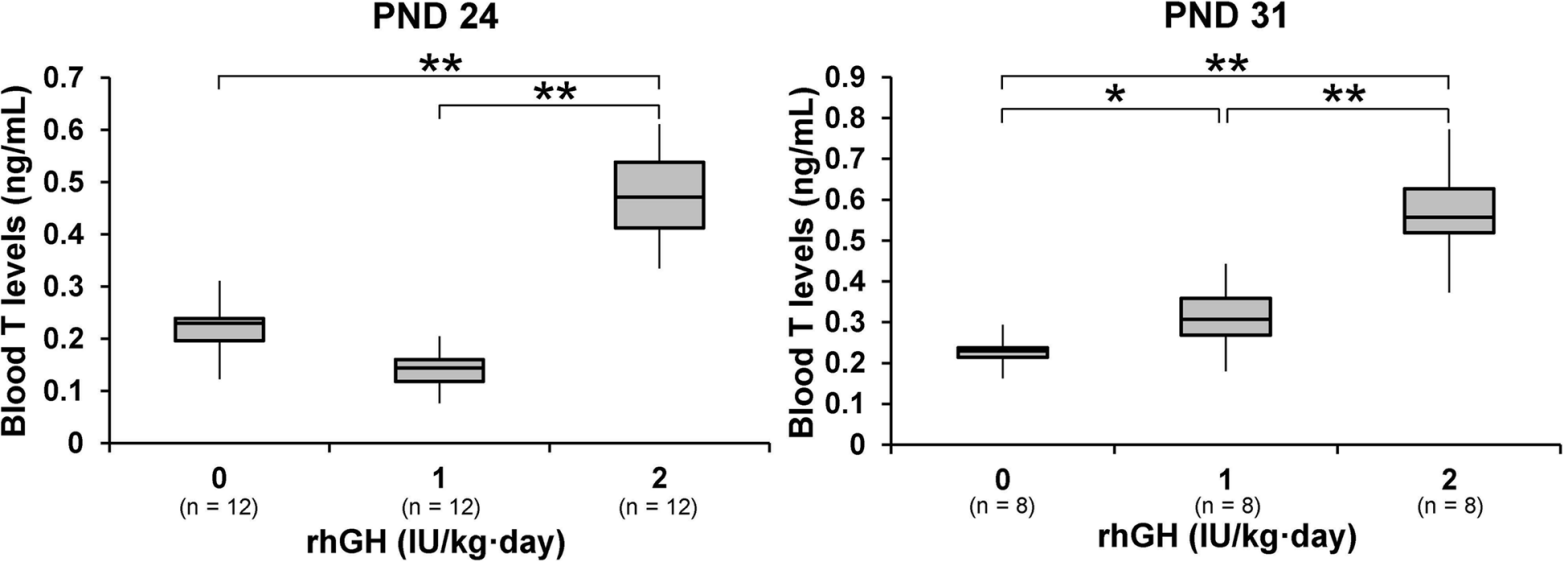
Effects of rhGH on blood testosterone levels in immature male rats. ELISA for blood testosterone levels on PNDs 24 (**A**) and 31 (**B**). * and **, significantly different from control rats by Mann-Whitney U test at *p* < 0.05 and 0.01, respectively

On PND 24, quantitative image analysis of HSD17B(+) immunoreactivity in testes revealed a significant increase in the number of LCs in 1 and 2 IU/kg rhGH rat testes. LCs number of 2 IU/kg rhGH rats was significantly higher than 1 IU/kg rhGH rats. The size of the HSD17B(+) LCs was significantly increased in 1 and 2 IU/kg rhGH rats. On PND 31, the size of the HSD17B(+) LCs of 1 and 2 IU/kg rhGH rats was significantly larger than those of controls, and the size of HSD17B(+) LCs of 2 IU/kg rhGH rats were significantly larger than those of 1 IU/kg rhGH rats. On PND 24 and 31 the interstitial cell number of rhGH-treated rat testes was not significantly different from those of controls (Fig. 3; Supplementary Fig. 2). In the HSD3B immunofluorescence labeling of testes from rhGH rats on PND 24, both the number of HSD3B(+) LCs and their size was significantly increased compared with the control (Fig. 3; Supplementary Fig. 3). However, the total number of interstitial cells from rhGH rat testes was not different from control on PND 24. In the RT-qPCR analysis, On PND 24, the mRNA levels of *luteinizing hormone/choriogonadotropin receptor* (*Lhcgr*) and *Hsd17b3* of 2 IU/kg rhGH rats were significantly higher than those of controls and 1 IU/kg rhGH rats. On PND 31, *Lhcgr* and *Hsd17b3* mRNA levels of 1 and 2 IU/kg rhGH rats were significantly higher than those of controls and the *Lhcgr* and *Hsd17b3* mRNA levels of 2 IU/kg rhGH rats were significantly higher than 1 IU/kg rhGH rats. The *steroidogenic factor 1*, (*Sf1*) mRNA levels of 1 and 2 IU/kg rhGH rats were significantly higher than those of controls on PND 24 and 31. *Star* mRNA levels of 1 and 2 IU/kg rhGH rats were significantly higher than those of controls on PND 24 but PND 31. The *cytochrome P450 family 11 subfamily A member 1* (*Cyp11a1*) mRNA levels of 1 and 2 IU/kg rhGH rats were significantly higher than those of controls on PND 31 but PND 24. On PND 24 and 31, *Cyp17a1* mRNA levels of 1 and 2 IU/kg rhGH rats were significantly higher than those of controls. On PND 24 and PND 31, the *Cyp19a1*, and *hydroxy-delta-5-steroid dehydrogenase 3 beta- and steroid delta-isomerase 1* (*Hsd3b1*) mRNA levels of rhGH-treated rats were not significantly different from those of controls (Fig. 4). On PND 24, serum testosterone levels of 2 IU/kg rhGH rats were significantly higher than those of controls and 1 IU/kg rhGH rats. On PNDs 31, serum testosterone levels of 1 and 2 IU/kg rhGH rats were significantly higher than those of controls. Serum testosterone levels of 2 IU/kg rhGH rats were significantly higher than 1 IU/kg rhGH rats (Fig. 5).

### Changes in hypothalamic *Kiss1* and *Gnrh1* and pituitary *Lhb* mRNA and circulating LH levels

**Fig. 6:**
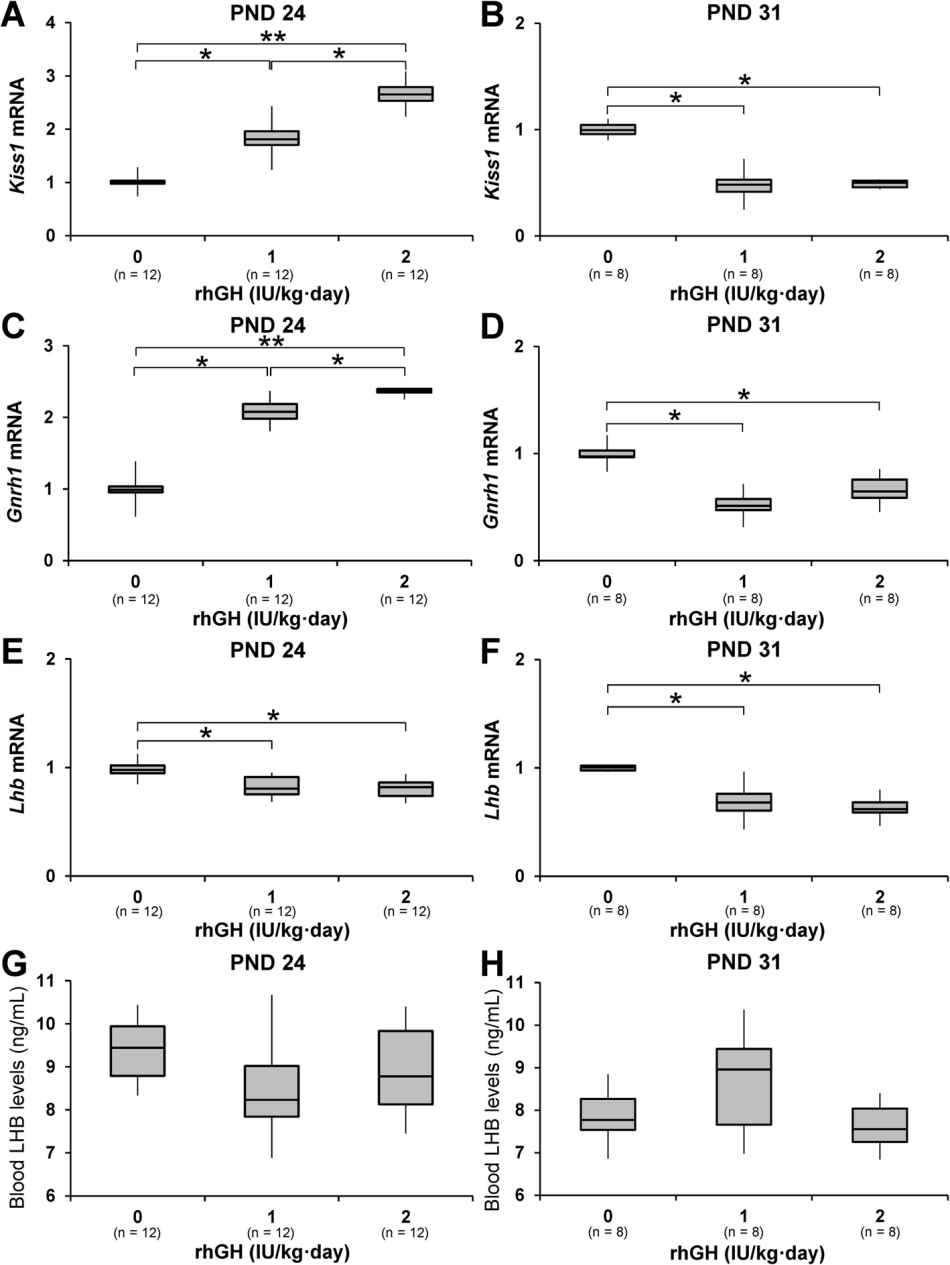
The effects of rhGH on hypothalamic *Kiss1* and *Gnrh1* and pituitary *Lhb* mRNA and blood LH levels. **A**-**F** RT-qPCR results for *Kiss1* and *Gnrh1* mRNA in the hypothalamus and pituitary *Lhb* mRNA levels on PNDs 24 and 31. **G**, **H** ELISA for blood LH levels on PNDs 24 and 31. * and **, significantly different from control rats by Mann-Whitney U test at *p* < 0.05 and 0.01, respectively

On PND24, hypothalamic *Kiss1* and *Gnrh1* mRNA levels of rhGH rats were significantly higher than those of control rats, the hypothalamic *Kiss1* and *Gnrh1* mRNA levels of 2 IU/kg rhGH rats were significantly higher than those of 1 IU/kg rhGH rats on PND 24. On PND 31, the hypothalamic *Kiss1* and *Gnrh1* mRNA levels of 1 and 2 IU/kg rhGH rats were significantly lower than those of controls. Pituitary *Lhb* mRNA levels of 1 and 2 IU/kg rhGH rats were significantly lower than those in the control rats on PNDs 24 and 31. No significant differences were found in circulating LH levels between the rhGH rats and control animals on PND 24 or 31 (Fig. 6).

### Changes in circulating IGF1 and mRNA levels in the hypothalamus, liver, and testes

**Fig. 7:**
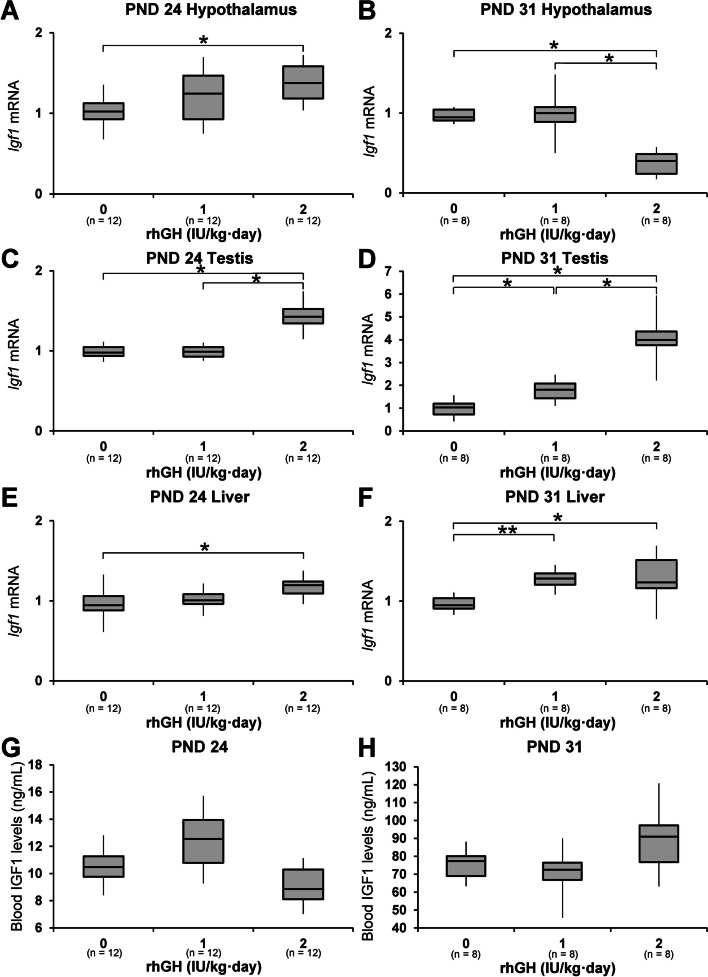
The effects of rhGH on blood IGF1 levels and *Igf1* mRNA levels in the hypothalamus, liver, and testes of immature rats on PNDs 24 and 31. **A**-**F** *Igf1* mRNA levels in the hypothalamus, liver, and testes on PNDs 24 and 31. **G**, **H** ELISA for blood IGF1 on PNDs 24 and 31. *, significantly different from control rats by Mann-Whitney U test at *p* < 0.05

On PND 24, the hypothalamic *Igf1* mRNA levels in 2 IU/kg rhGH rats were significantly higher than those in the control animals. On PND 31, the hypothalamic *Igf1* mRNA levels of 2 IU/kg rhGH rats were significantly lower than those of controls and 1 IU/kg rhGH rats. On PND 24, the liver *Igf1* mRNA levels of 2 IU/kg rhGH rats were significantly higher than those of controls. On PND 31, the liver *Igf1* mRNA levels of 1 and 2 IU/kg rhGH rats were significantly higher than those of controls. Blood IGF1 levels of the rhGH rats were not different significantly from those of the control rats on PND 24 or 31. On PND 24, the testicular *Igf1* mRNA levels of 2 IU/kg rhGH rats were significantly higher than those of the controls and 1 IU/kg rhGH rats. On PND 31, the testicular *Igf1* mRNA levels of 1 and 2 IU/kg rhGH rats were significantly higher than those of the controls, and the *Igf1* mRNA levels of 2 IU/kg rhGH rats were significantly higher than those of 1 IU/kg rhGH rats (Fig. 7).

### Changes in *Igf1* expression and steroidogenesis in LCs *in vitro*

**Fig. 8:**
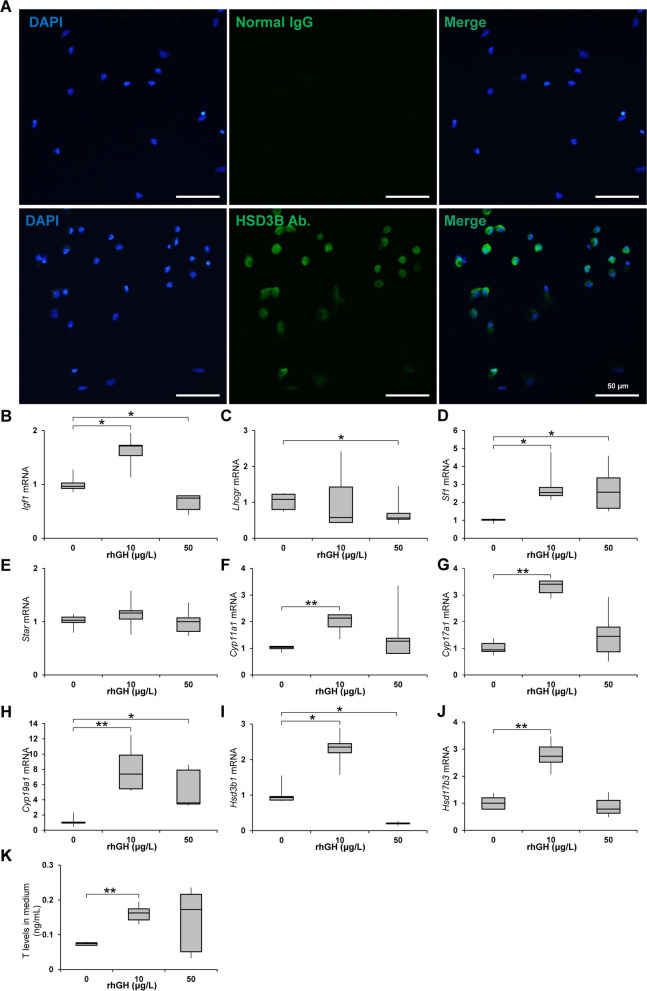
Changes in steroidogenesis in LCs *in vitro*. **A** Purity of LCs as shown by immunocytochemistry for HSD3B. **B**–**J** RT-qPCR results for *Igf1*, *Lhcgr*, *Sf1*, *Star*, *Cyp11a1*, *Cyp17a1*, *Cyp19a1*, *Hsd3b1*, and *Hsd17b3* mRNA in rhGH-treated LCs *in vitro* (*n *= 5). **K** ELISA for testosterone in the culture medium of LCs after 48 h of rhGH treatment (*n *= 5). * and **, significantly different from control rats by Mann-Whitney U test at *p* < 0.05 and 0.01, respectively

In the immunocytochemical staining, 96.3% of the isolated interstitial cells were positive for HSD3B. The *Cyp11a1* and *Hsd17b3* mRNA levels in LCs treated with 10 μg/L (0.03 IU/L) rhGH were significantly higher than those in the control cells, but the levels in cells treated with 50 μg/L (0.15 IU/L) of rhGH did not differ from the control cells. Compared to those of control cells, *Igf1* mRNA levels were significantly higher in LCs treated with 10 μg/L (0.03 IU/L) rhGH but significantly lower in LCs treated with 50 μg/L (0.15 IU/L) rhGH. *Lhcgr* mRNA levels of 50 μg/L (0.15 IU/L) rhGH-treated LCs were significantly lower than those of controls. *Sf1* and *Cyp19a1* mRNA levels in LCs treated with 10 or 50 μg/L (0.03 or 0.15 IU/L) rhGH were significantly higher than those of control cells. *Hsd3b1* mRNA levels were significantly higher in LCs treated with 10 μg/L (0.03 IU/L) rhGH but *Hsd3b1* mRNA levels of 50 μg/L (0.15 IU/L) rhGH treated LC were significantly lower than those of controls. *Star* mRNA levels were not different significantly between the rhGH-treated cells and the control. In the spent media, testosterone levels were significantly higher than the control in LCs treated with 10 μg/L (0.03 IU/L) rhGH but not in those treated with 50 μg/L (0.15 IU/L) rhGH (Fig. 8).

## Discussion

### Effect of rhGH on spermatogenesis and steroidogenesis in immature rats

Correlations between GH and sexual maturation have been reported in both sexes [28]. In immature male rats, puberty was advanced, together with an increase in total body weight and reproductive organ weight following rhGH administration [29]. In this study, the testis weights of rhGH rats were lower than those of control rats on PND 24, although that difference was mitigated on PND 31. Similarly, in immature male dogs, a high dose of rhGH induced atrophy of the testes and accessory organs [30]. GH can induce apoptosis and atretic changes in the ovaries and testes of mammals, including humans, by activating the PI3K-Akt pathway [31]. Though no explanation for the reduced testis weights in rhGH rats during the early prepubertal period has been confirmed, high doses of rhGH in the early prepubertal period might transiently reduce testis development in a way that is impermanent and recoverable. In histology, the diameter and luminal areas of the seminiferous tubules were visibly increased in 2 IU/kg rhGH rats on PND 31 together with the spermatogenic markers mRNA levels, indicating that rhGH activated spermatogenesis in the immature male rats. In immature rats, GH treatment induced testicular growth and germ cell differentiation [12]. In rhGH rats, blood testosterone levels were higher than control rats on PNDs 24 and 31. Similarly, rhGH injection stimulated testosterone synthesis in immature and adult rodents [32]. Given that circulating LH levels did not differ between the control and rhGH rats, the activation of androgen production might indicate that rhGH has a direct effect on LCs. In cultures of LCs, rhGH directly activated steroidogenesis [12]. In rat testes, HSD3B(+) PLCs are typically observed on PND 21 before they increase in number and become HSD17B(+) immature LCs that go on to differentiate into functional adult LCs [33]. In light of the observed mRNA levels of steroidogenic pathway genes, increases in the number and mean size of HSD17B(+) LCs in rhGH rat testes on PNDs 24 and 31, and the number and mean size of HSD3B(+) LCs in 2 IU/kg rhGH was higher than 1 IU/kg rhGH rat testes, prepubertal rhGH administration might have accelerated the functional differentiation of PLCs into immature, testosterone-producing LCs. In the mammalian testes, GH and IGF1 modulate the proliferation and steroidogenesis of LCs [12, 34]. In HSD3B(+) PLCs isolated on PND 21 rats, rhGH treatment increased the steroidogenic pathway gene mRNA and testosterone secretion. These suggest that rhGH treatment directly activates steroidogenesis in PLCs, which is consistent with the previous studies [35]. The increase in circulating testosterone found in rhGH rats on PNDs 24 and 31 might be attributable to the potentiation of steroidogenic differentiation of PLCs to testosterone-producing LCs, leading to activation of the spermatogenesis.

### rhGH triggered changes in kisspeptin, GnRH, and LH in immature male rats

During the prepubertal period, kisspeptin activates the HPG axis and increases sex steroids [36, 37]. In male rats, hypothalamic kisspeptin and GnRH concentrations were elevated after PND 7 [38]. In prepubertal mice, rats, and ewe, rhGH administration potentiated hypothalamic kisspeptin and GnRH production [39]. In adult mice, hypothalamic *Kiss1* and *Gnrh1* mRNA were downregulated by sex steroids [39, 40]. In this study, hypothalamic *Kiss1* and *Gnrh1* mRNA levels in the rhGH rats were higher than those in the control rats on PND 24, when kisspeptin is elevated in male rats. Circulating testosterone levels in the 2 IU/kg rhGH rats were higher than those in the control rats on PNDs 24 and 31. Although the circulating LH levels of the rhGH rats did not differ from those of the control rats on PND 24 or 31, the pituitary *Lhb* mRNA levels in the rhGH rats were lower than those in the control rats on PNDs 24 and 31, suggesting that elevated testosterone levels provide negative feedback for the expression of pituitary *Lhb*.

### rhGH triggered IGF1 changes in the hypothalamus, liver, and testes of immature rats

**Fig. 9:**
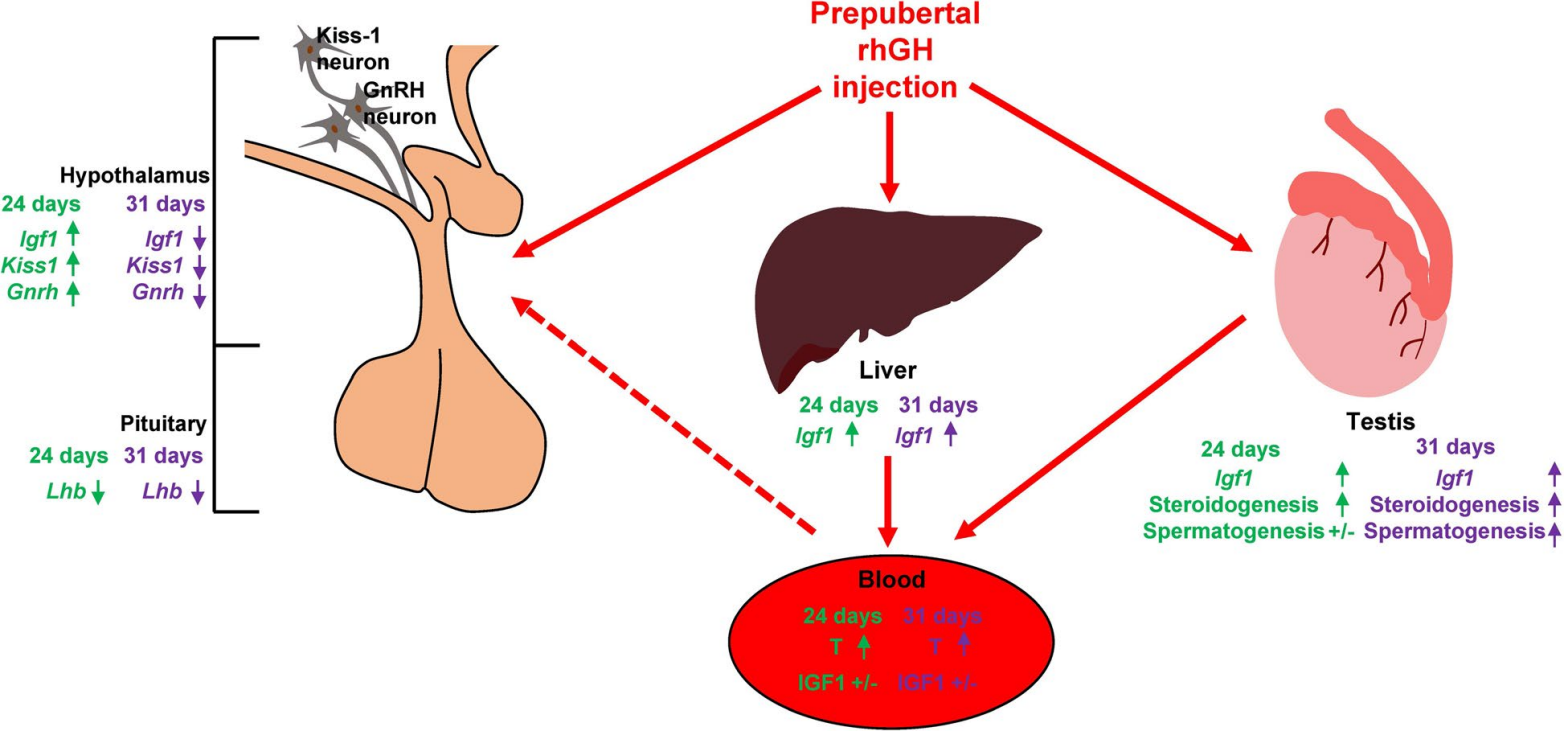
Local increase in IGF1 levels in the testes might be an important mediator for gonadal activation triggered by rhGH in immature rats. Changes in kisspeptin, GnRH, LH, IGF1, and testosterone in the hypothalamus-pituitary-testis axis following daily injections of rhGH from PND 21 to 23 or PND 21 to 30 are summarized. Testicular steroidogenesis, circulating testosterone, and spermatogenesis were higher than control levels in the rhGH rats on PNDs 24 and 31. Hypothalamic *Kiss1, Gnrh1*, and *Igf1* mRNA levels were also higher than control levels in rhGH rats on PND 24, but they became lower than the control levels by PND 31. No significant change in circulating LH levels was observed on PND 24 or 31 in the rhGH rats. In prepubertal male rats, rhGH administration increased testicular but not systemic IGF1 levels, which potentiated testosterone production in LCs and thus increased circulating testosterone, stimulating sexual maturation and spermatogenesis

IGF1 mediates GH-dependent and GH-independent anabolism and growth [41, 42]. In rodents and primates, hypothalamic IGF1 expression increases at puberty, which activates kisspeptin-GnRH neurons [3, 15]. In human and mouse brains, hypothalamic GnRH neurons express the IGF1 receptor [43, 44]. In prepubertal female rats, a cerebroventricular infusion of IGF1 stimulated the secretion of GnRH and might advance the onset of puberty [15, 18]. In prepubertal male rats, a central infusion of IGF1 antiserum delayed pubertal development [45]. Altogether, brain IGF1 is an important factor in the initiation of puberty. In hypothalamus, sex steroids activate the GH-IGF1 axis, accelerating the growth and maturation of reproductive organs for puberty [46]. Together, increased hypothalamic *Igf1* mRNA in rhGH rats on PND 24 might be attributable to the elevated testosterone, as well as the direct action of rhGH on hypothalamic *Igf1* expression. In contrast, on PND 31, hypothalamic *Igf1* mRNA levels in 2 IU/kg rhGH rats were lower than those in the control rats, which could be a result of negative feedback from elevated androgens in 2 IU/kg rhGH rats. Stimulation of IGF1 production in liver has been considered to be a major effect of GH [47]. In the rhGH rats, liver *Igf1* mRNA levels were increased without visible increase in blood IGF1 levels. Testicular *Igf1* mRNA levels of rhGH rats were higher than those of control rats on both PND 24 and 31. Similarly, in immature hypophysectomized rats, rhGH increased testicular but circulating IGF1 levels [17, 48]. Given that IGF1 and cognate receptor are expressed in germ cells, LCs and Sertoli cells [49], intratesticular IGF1 could regulate various aspects of testicular function in both autocrine and paracrine manners. In rodents, IGF1 can promote the proliferation, maturation, and steroidogenesis of LCs by means of para- and autocrine action [50]. In mouse LCs, the expression of LH receptor and response to LH are potentiated by IGF1 [51]. In prepubertal male rats, rhGH treatment increased the number and size of HSD3B(+) and HSD17B(+) LCs in testes. In isolated HSD3B(+) PLCs, rhGH treatment increased the *Igf1* mRNA. Therefore, testicular increases in IGF1 could mediate functional differentiation of testosterone-producing LCs in rhGH rats, and the increased circulating testosterone in rhGH rats may be due to the increased LH response in LCs by the testicular IGF1. The elevated testicular IGF1 levels might be responsible for the activation of testosterone production and spermatogenesis (Fig. 9). In gonadotropin-independent precocious puberty (GIPP), sexual maturation is induced by sex steroids that increase through a gonadotropin-independent mechanism such as testotoxicosis, tumors, or environmental hormones [52]. The elevation of testicular IGF1 through prepubertal administration of rhGH could thus evoke the early onset of sexual maturation without increasing circulating LH levels, resembling GIPP.

## Conclusion

In prepubertal male rats, high dose rhGH administration increased testicular IGF1 levels, which may potentiate testosterone production in LCs and spermatogenesis. In terms of clinical application, to prevent the adverse effects of rhGH in prepubertal children, reduction of rhGH dosage can be considered to avoid precocious puberty.

## Supplementary Information

**Additional file 1: Supplementary Figure 1.** Hematoxylin and eosin staining of testes from rhGH rats on PNDs 24 and 31.

**Additional file 2: Supplementary Figure 2.** Immunocytochemistry for HSD17B in the testes of rhGH rats on PNDs 24 and 31.

**Additional file 3: Supplementary Figure 3.** Immunofluorescence for HSD3B in the testes of rhGH rats on PND 24. The blue color in the nucleus shows DAPI staining, the red color shows HSD3B staining.

## Data Availability

The data sets used and/or analysed during the current study are available from the corresponding author on reasonable request.

## Abbreviations

% (v/v): Percent (volume per volume)
°C: Degree Celsius
μm: Micrometer
a.m.: Ante meridiem
CO_2_: Carbon dioxide
*Cyp11a1*: *Cytochrome P450 family 11 subfamily A member 1*
*Cyp17a1*: *Cytochrome P450 family 17 subfamily A member 1*
*Cyp19a1*: *Cytochrome* *P450 family 19 subfamily A member 1*
ELISA: Enzyme-linked immunosorbent assay
GH: Growth hormone
GIPP: Gonadotropin-independent precocious puberty
GnRH: Gonadotropin-releasing hormone
*Gnrh1*: *Gonadotropin Releasing Hormone 1*
HPG axis: Hypothalamus-pituitary-gonad axis
hr.: Hour
HSD17b: 17β-hydroxysteroid dehydrogenase
*Hsd17b3*: *Hydroxysteroid 17β dehydrogenase 3*
Hsd3b: 3β-hydroxysteroid dehydrogenase
*Hsd3b1*: *Hydroxy-delta-5-steroid dehydrogenase, 3 beta- and steroid delta-isomerase 1*
Igf1: Insulin-like growth factor-1
IgG: Immunoglobulin G
IU: International units
kg: Kilogram
*Kiss1*: *KiSS-1 Metastasis Suppressor*
L: Liter
LCs: Leydig cells
LH: Luteinizing hormone
Lhb: Luteinizing hormone beta polypeptide
*Lhcgr*: *Luteinizing hormone/choriogonadotropin receptor*
mL: Milliliter
mRNA: Messenger RNA
PBS: Phosphate buffered saline
PCR: Polymerase chain reaction
PFA: Paraformaldehyde
PLC: Progenitor Leydig cell
PND: Postnatal day
*Prm*: *Protamine*
rhGH: Recombinant human growth hormone
RNA: Ribonucleic acid
*Rpl7*: *Ribosomal protein L7*
rpm: Revolutions per minute
RPMI: Roswell Park Memorial Institute medium
RT-qPCR: Real-time reverse transcription-polymerase chain reaction
*Sf1*: *Steroidogenic factor 1*
*Star*: *Steroidogenic acute regulatory protein*
T: Testosterone
*Tnp1*: *Transition protein 1*
*Tnp2*: *Transition protein 2*
μg: Microgram
